# Slot Self-Allocation Based MAC Protocol for Energy Harvesting Nano-Networks

**DOI:** 10.3390/s19214646

**Published:** 2019-10-25

**Authors:** Wan-Liang Wang, Chao-Chao Wang, Xin-Wei Yao

**Affiliations:** College of Computer Science and Technology Zhejiang University of Technology, Hangzhou 310023, China

**Keywords:** nano-networks, energy harvesting, self-allocation, MAC protocol, terahertz communications

## Abstract

Nano-networks are composed of interconnected nano-nodes and can enable unprecedented applications in various fields. Due to the peculiarities of nano-networks, such as high density, extremely limited energy and computational resources, traditional carrier-sensing based Media Access Control (MAC) protocols are not suitable for nano-networks. In this paper, a Slot Self-Allocation based MAC protocol (SSA-MAC) is proposed for energy harvesting nano-networks. Two transmission schemes for centralized and distributed nano-networks are designed, respectively. In centralized nano-networks, nano-nodes can only send packets to the nano-controller in their Self-Allocation Slots (SASs), while, in distributed nano-networks, nano-nodes can only receive packets from surrounding nano-nodes in their SASs. Extensive simulations were conducted to compare the proposed SSA-MAC with PHysical LAyer aware MAC (PHLAME), Receiver-Initiated Harvesting-aware MAC (RIH-MAC) and Energy Efficient Wireless NanoSensor Network MAC (EEWNSN). From the results, it can be concluded that the proposed SSA-MAC achieves better performance and can reduce the collision probability, while improving the energy efficiency of nano-networks.

## 1. Introduction

With the development of nanotechnology, nano-devices, e.g., nano-sensors, nano-batteries and nano-processors, are becoming a reality [1,2]. Nano-nodes assembled of these nano-devices show new functionalities and sensing abilities. For example, nano-nodes can be used in the human body to supervise the health parameters of people [3], as well as to detect and prevent harmful chemicals and bacteria [4]. However, a single nano-node can only provide limited sensing, computing and memorizing abilities due to its extremely small size and resulting constrained resources. Nano-networks composed of interconnected nano-nodes are developed to overcome these limitations, which can be implemented to enable novel applications, such as advanced health monitoring systems [4], planting monitoring systems [5], pollution prevention systems [6] and other industrial and consumer applications [7].

The nodes working in nano-networks are composed of many components in the nano-scale, which make the existing technologies in wireless sensor networks (WSNs) unsuitable for nano-networks. One critical problem in nano-networks is the communication method among nano-nodes. Due to the extremely limited size of nano-nodes, and the resulting small transceivers and antennas, ultra-high transmission frequency is imposed. Recently, nano-antennas based on graphene electronics are developed to radiate Terahertz (THz) band (0.1–10 THz) signals to address this problem [8,9]. In traditional WSN, nodes communicate at hundred Megahertz (MHz) to several Gigahertz (GHz), which is totally different with THz band. Moreover, new advancements in molecular and carbon electronics make the manufacturing of nano-components (including nano-transceivers, nano-processors, etc.) come true [10,11,12]. In the past years, some attempts have been made for nano-networks in the design of nano-components [10,13,14,15], physical layer models [16,17,18], communication protocols [19,20,21,22] and other applications [5,6,23].

However, it still requires lots of works to guarantee the communications in nano-networks. There are some challenges in the design of MAC protocols for nano-networks:

- The computational resource constraint. Because of the small size of nano-node, the actual nano-processor is limited in size and, thus, in the number of transistors. Therefore, MAC protocols for nano-networks should be simple and easy to be implemented.
- The very limited energy storage, and the implementation of energy harvesting systems. The small size of nano-node also limits the energy storage [24]. Hence, energy harvesting systems (e.g., zinc-oxide nano-wires [12] and piezoelectric nano-generators [25,26]) are proposed to be implemented in nano-nodes to capture energy from the environment [27]. However, because of the restrictions of technical conditions, the energy harvesting speed is still not high enough; hence, MAC protocols should be energy efficient and have the ability to adapt to the energy fluctuations of nano-node.
- Due to the expectedly very high nano-node density, collisions could happen during transmissions and result in transmission failures. Hence, novel MAC protocols are needed to allocate the channel resources and to coordinate concurrent transmission among nano-nodes.
- Traditional carrier-sensing based MAC protocols are not suitable for nano-networks. Due to the energy constraint of nano-node and high path loss of THz band, it is currently not feasible to generate high-power continuous carrier signals at THz frequencies [19]. Hence, pulse-based modulation schemes are recommended [28,29]. Therefore, the peculiarities of THz signals and pulse-based modulation methods should be taken into consideration for MAC protocols.

To address the above challenges, reduce the collision probability during data transmissions among nano-nodes, and improve the energy efficiency, a Slot Self-Allocation based MAC protocol (SSA-MAC) is proposed, which can be utilized both in centralized and distributed nano-networks. In the proposed SSA-MAC protocol, a time frame is divided into several time slots. Nano-nodes can choose one time slot as their Self-Allocated Slots (SASs) by their own identifications (IDs), and set the other time slots as Sleep Slots (SSs). In centralized nano-networks, nano-nodes can only send packets to the nano-controller at their own SASs. In distributed nano-networks, nano-nodes can only receive packets from others in their own SASs. When a nano-node wants to send packets to a specific receiving nano-node, it calculates the receiving nano-node’s SAS by its ID and sends the packet at the corresponding time slot. The main contributions of this paper are as follows:

- Aiming at the high density of nano-nodes and resulting high collision probability during data transmissions in nano-networks, a slot self-allocation based MAC protocol is proposed to reduce transmission failure probability and improve energy efficiency.
- For different nano-network structures, i.e., centralized and distributed nano-networks, different transmission schemes are designed, respectively. Furthermore, the performances of the two transmission schemes are analyzed, including energy consumption, packet delay and throughput.
- In the simulations, the performance of SSA-MAC is simulated with different values of parameters. According to the results, the proposed SSA-MAC outperforms PHLAME, RIH-MAC and EEWNSN MAC protocols.

The remainder of this paper is organized as follows. The system model is introduced in Section 3, including the adopted Time Spread-On Off Keying modulation scheme (TS-OOK), and centralized and distributed nano-network structures. In Section 4, the details of SSA-MAC are presented. The performance analysis is provided in Section 5. In Section 6, extensive simulations are conducted to compare the proposed SSA-MAC with PHLAME, RIH-MAC and EEWNSN MAC protocols. Finally, we conclude in Section 7.

## 2. Related Work

At present, there are some scholars studying MAC protocols for nano-networks. In [19], the authors proposed a PHysical LAyer aware MAC protocol (PHLAME) based on Rate Division Time Spread-On Off Keying (RD TS-OOK) modulation method. In PHLAME, nano-nodes can adopt different symbol rates for different transmissions and types of packets, so that the interference among nano-nodes can be effectively reduced. In [20,30], the authors proposed a Receiver-Initiated Harvesting-aware MAC protocol (RIH-MAC) for both centralized and distributed nano-networks. In RIH-MAC, the receiver broadcasts Ready To Receive (RTR) packets periodically, and any surrounding nano-nodes that receive the RTR packet will compete with the others to send packets to the corresponding receiver with sufficient energy. The authors presented an energy-aware MAC protocol for Grid Based nano-networks (GB-MAC) in [31]. This protocol can improve the performance of nano-networks by allocating different channels and active time slots to the different nano-nodes. The authors of [32] presented an Energy Efficient Wireless NanoSensor Network MAC protocol (EEWNSN), where nano-nodes can only send packets to the nano-controller during allocated time slots. In other networks, to address the limitation on available resources, state-free gradient-based and redundancy-based transport schemes are proposed to reduce the delay and improve the throughput [33,34]. Furthermore, to reduce the collision probability and improve energy efficiency of nano-nodes, the ideas of time division and sleep-wakeup methods in traditional sensor networks can be considered [35,36,37].

However, there are several disadvantages of the above MAC protocols: (i) In PHLANME, the protocol needs a handshake process to confirm the symbol rate before transmitting, and the energy constraint and energy harvesting process are not considered. Moreover, due to the ultra high density of nano-networks, the collisions could still happen and result in retransmissions between nano-nodes, which consumes extra energy of nano-nodes. (ii) In RIH-MAC, the periodic receiving requirements could introduce collisions among nano-nodes and waste energy when there is no packet needed to be sent. (iii) In GB-MAC, it is difficult to obtain the locations of all the nano-nodes in all nano-networks, and, thus, it only suits grid-based nano-networks. (iv) In EEWNSN MAC protocol, nano-nodes have to communicate with the nano-controller to allocate transmitting slots, which cannot be implemented in distributed nano-networks. (v) The MAC protocols in other networks and traditional sensor networks cannot be utilized in nano-networks directly due to the resources constraints of nano-nodes and peculiarities of THz band.

## 3. System Model

In this section, the adopted modulation scheme named TS-OOK is presented [38]. Moreover, two different nano-network structures are described, namely centralized and distributed structures.

### 3.1. TS-OOK Modulation Scheme

On the one hand, due to the size and resulting energy limitation of nano-nodes, they cannot provide high transmission power. On the other hand, nano-nodes have to communicate at THz band, which suffers severe spreading loss and molecular absorption loss [39]. Hence, continuous carrier signal techniques cannot be used in nano-networks; only pulse-based modulation schemes can be considered [19]. In this paper, a pulse-based modulation scheme named TS-OOK (regarded as the most promising modulation method for nano-networks) is adopted.

**Figure 1:**
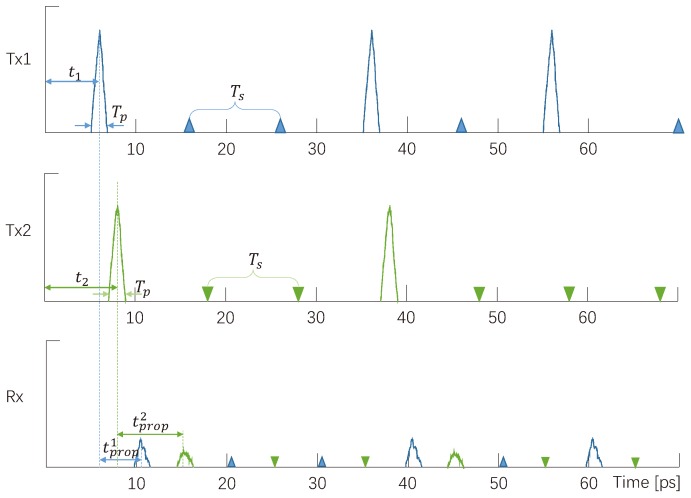
Examples of TS-OOK modulation scheme.

In TS-OOK, when a nano-node needs to send a packet, the logical “1” in the packet is modulated as a femto-second-long pulse, logical “0” is modulated as silence. As shown in Figure 1, there are two transmitting nano-nodes, $Tx_{1}$ and $Tx_{2}$, sending packets to the receiver Rx simultaneously. The sequence sent by $Tx_{1}$ is “1001010” and the sequence sent by $Tx_{2}$ is “1001000”. Due to the high spreading loss and molecular absorption loss, the received pulse power would be much smaller than the transmitting power. The time interval between two symbols named symbol duration $T_{s}$ is fixed so that every nano-node and nano-controller can sync to demodulate the signals. $T_{s}$ is much longer than the pulse duration $T_{p}$, so that the receiver can have enough time to diminish the effects of noise and interference, and detects signals. Moreover, since $T_{s}$ is much longer than $T_{p}$, the receiver does not need to listen to the channel all the time, i.e., it can receive signals from other nano-nodes after receiving the pulse.

There are two advantages of TS-OOK. First, the nano-node only consumes energy while transmitting logical “1”, but spends no energy while transmitting logical “0”. Second, it can reduce the interference of nano-networks to improve the network performance. Moreover, an improved RD TS-OOK modulation scheme is proposed in [19], which allows nano-nodes to use different symbol rates (defined as $T_{s}/T_{p}$) for different nano-nodes and different types of packets. This can further reduce interference and collision probability. However, a transmitting nano-node has to exchange the symbol rates with the receiving nano-node by using a handshake process before transmitting, which needs extra computation and energy. However, it needs strict time synchronization among nano-nodes, which is still a challenge in nano-networks. Hence, we choose the TS-OOK modulation scheme for the proposed SSA-MAC protocol.

### 3.2. Network Structure

**Figure 2:**
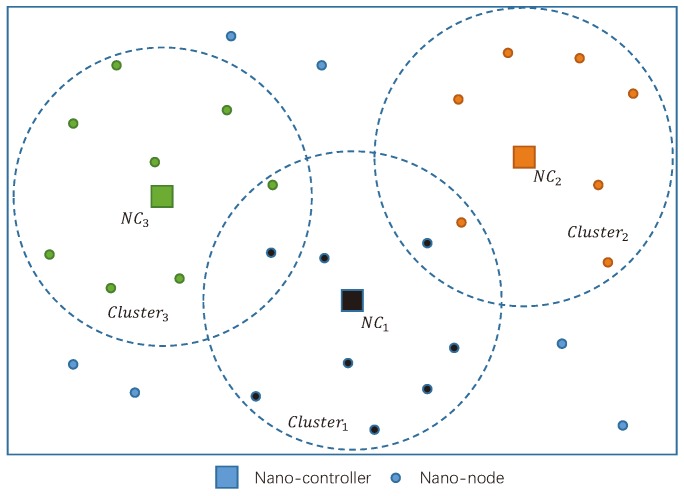
An example of centralized nano-network.

In general, the structures of nano-networks can be divided into two types, namely centralized and distributed nano-networks. In centralized nano-networks, there are two basic kinds of nano-devices, i.e., nano-node and nano-controller. Nano-nodes are responsible for collecting data from the environment and send the data to their closest nano-controller. All the nano-nodes are equipped with energy harvesting units, which can capture energy from the environment. More details of energy harvesting systems can be found in [12,25,26,27]. Nano-controllers are not selected from nano-nodes. Nano-controllers are fixed in the networks and have enough power to manage a number of nano-nodes within its coverage, and can send the collected data to nano-gateways or macro networks. An example of centralized nano-network is presented in Figure 2. There are three nano-controllers dominating three clusters of the network.

**Figure 3:**
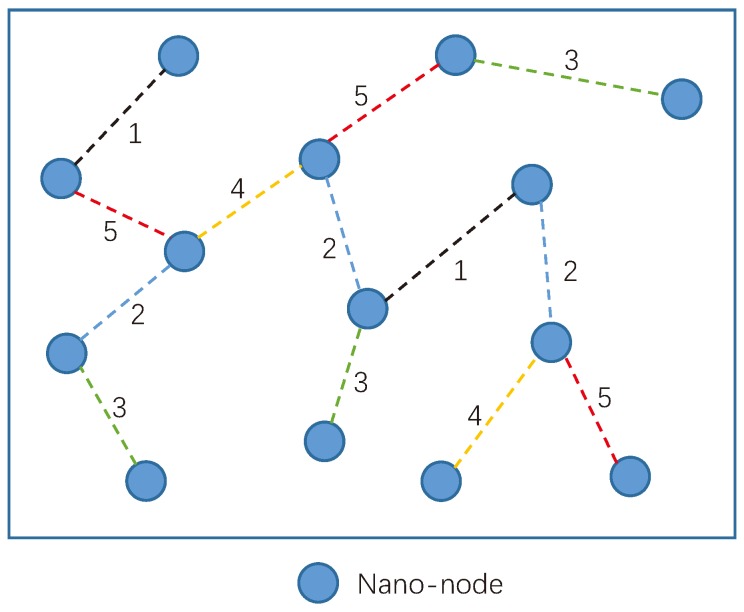
An example of distributed nano-network.

In distributed nano-networks, communications between nano-nodes are self-organized. Nano-nodes make decisions by their own and cooperate with their neighboring nano-nodes within transmission range. Figure 3 presents an example of distributed nano-network. Due to the self-organized communication scheme adopted by the nano-nodes, communications among nano-nodes may occur at different time. The numbers in the figure represent the time of different communications.

The proposed SSA-MAC can be implemented in both nano-network structures. However, the specific communication schemes for these two nano-network structures are different, which is explained in the following section.

## 4. The Proposed SSA-MAC Protocol

In SSA-MAC, a time frame is divided into several time slots; every nano-node can allocate one slot as SAS by its own ID. In centralized nano-networks, nano-nodes only send data to the nano-controller in their own SASs. In distributed nano-networks, nano-nodes only receive data from other nano-nodes in their own SASs. When a nano-node wants to send data to another nano-node, it calculates the receiver’s SAS, and then sends data in the corresponding time slot. In this section, the time slot division and allocation methods are described, as well as the transmission schemes for different nano-network structures.

### 4.1. SAS Division and Allocation Methods

**Figure 4:**
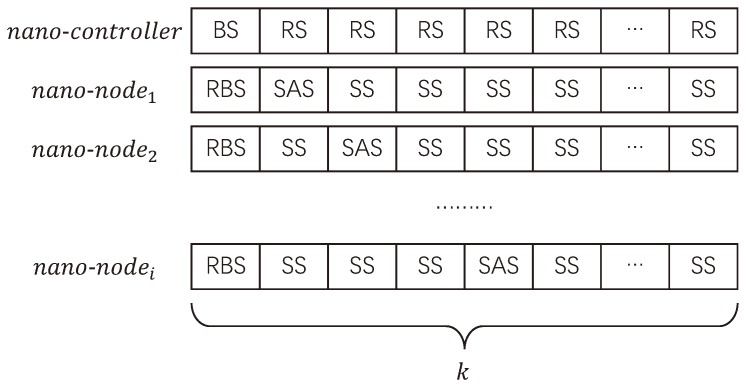
An example of time slots division and allocation in SSA-MAC.

In the SSA-MAC, nano-nodes can allocate SASs by themselves according to their own IDs. An example of time slots division and allocation is presented in Figure 4. For nano-controller, there are two kinds of time slots, one is Broadcast Slot (BS) for broadcasting packets to nano-nodes to update the information (e.g., time and transmission protocols) and functions of nano-nodes and the other is Receive Slot (RS) for receiving packets from nano-nodes.

For nano-nodes, there are three kinds of time slots, the first one is Receive Broadcast Slot (RBS). Since all the nano-node in SSA-MAC do not run handshake process before transmissions, they need to operate in a time-synchronous manner. Hence, one of the functions of RBS is for time synchronization. In centralized nano-networks, the nano-node can receive time packets from nano-controller. In distributed nano-networks, the nano-nodes can execute some time synchronization algorithms, e.g., Network Time Protocol (NTP) [40], or receive time packets from a special nano-controller. However, it is still a very challenging problem in distributed networks and is worth studying. Moreover, RBS can be also used for neighbor discovering and function updating. The second one is the SAS for sending packets to the nano-controller in centralized nano-networks and receiving packets from the other nano-nodes in distributed nano-networks. The third one is sleep slot for harvesting energy and collecting data from the environment. Furthermore, the SSs will transfer into transmitting slots in distributed nano-networks when nano-nodes need to send packets to the other nano-nodes.

A nano-node can allocate its own SASs by

(1) $$S_{AS}=\left(ID\;\;\mathrm{mod}\;\;k\right)+1,$$

where $S_{AS}$ is the number of SAS, ID is the unique identification of nano-node, and mod refers to the modulus operation. As the example in Figure 4 shows, the first time slot of each nano-node is RBS, the second time slot of nano-${\mathrm{sensor}}_{1}$ is SAS, and the third time slot of nano-${\mathrm{sensor}}_{2}$ is SAS. *k* is the number of time slots that a time frame can be divided into which can be obtained as follows:

(2) $$k=\frac{T_{frame}}{T_{slot}}+1,$$

where $T_{frame}$ is the length of time frame and is set larger than the time of harvesting enough energy for one packet transmission, so that it can be guaranteed that the nano-nodes harvest enough energy for transmitting after a time frame. $T_{frame}$ can be expressed as follows:

(3) $$T_{frame}\geq \frac{E_{Data}^{T}+E_{ACK}^{R}}{S_{harv}},$$

where $S_{harv}$ is the energy harvesting speed of nano-node and $E_{Data}^{T}$ and $E_{ACK}^{R}$ are the energy for sending a data packet and receiving an ACKnowledgement (ACK) packet, respectively. $E_{Data}^{T}$ and $E_{ACK}^{R}$ can be obtained by

(4) $$E_{Data}^{T}=\eta_{Data}B_{Data}E_{p},$$

(5) $$E_{ACK}^{R}=\eta_{ACK}B_{ACK}E_{p},$$

where $B_{Data}$ and $B_{ACK}$ are the sizes in bit of data packet and ACK packet, respectively. $E_{p}$ is the energy for one pulse. $\eta_{Data}$ and $\eta_{ACK}$ are the ratios of symbol “1” in data packet and ACK packet, respectively. $T_{slot}$ is the length of the time slot, which depends on the nano-node quantity in the nano-network:

(6) $$T_{slot}=\frac{T_{frame}}{N_{node}},$$

where $N_{node}$ is the nano-node quantity. However, $T_{slot}$ cannot be infinitely small. It should exceed two times of the time of sending a data packet and receiving an ACK packet, which can be expressed as

(7) $$T_{slot}>2\left(B_{data}+B_{ACK}\right)T_{s}.$$

**Figure 5:**
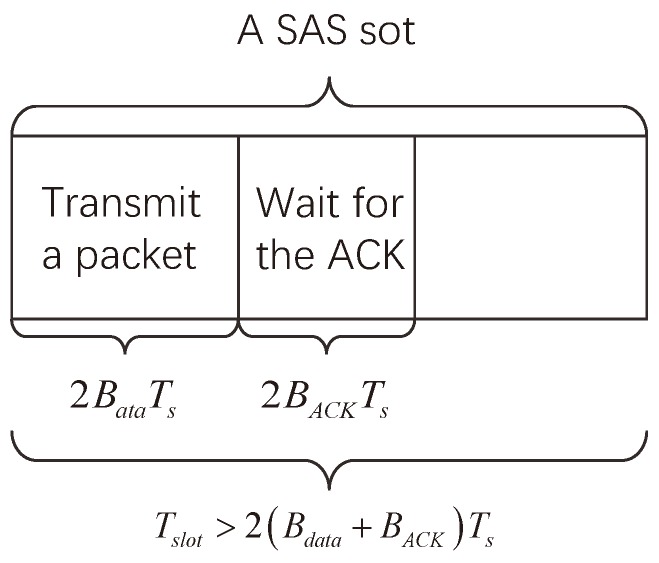
An example of SAS slot.

Above all, one example of SAS slot is given in Figure 5.

### 4.2. Transmission Scheme in Centralized Nano-Networks

In centralized nano-networks, nano-nodes collect information from the environment and send the data to the nano-controller, and then the nano-controller sends the data to nano-gateways or macro network. The centralized structure is easy to implement and has strong expansibility; hence, it is used widely in many nano-network applications [32]. Compared with nano-nodes, nano-controller has enough energy and stronger computation abilities. Therefore, it can keep receiving from nano-nodes. The operations of transmissions in centralized nano-networks are as follows:

- In the initial stage, nano-nodes operate the time slot allocation procedures, they set their first time slots as RBSs, and allocate one SAS by their own IDs according to Equation (1), and set the other time slots as SSs.
- In RBSs, nano-nodes receive broadcast packets from the nano-controller for function updating and time synchronization.
- In SSs, nano-nodes do not send or receive packets, but energy harvesting and data collecting systems still work.
- Before transmitting data packets, nano-nodes check whether the energy is enough or not. If yes, they send the data packet in their corresponding time slots. Otherwise, they wait for the next time frame. According to Equation (3), the length of time frame is set larger than the time of harvesting enough energy for transmitting one data packet and receiving one ACK/NACK packet.
- When a nano-node collects enough data and need to send it to the nano-controller, it waits until its own SAS and sends the data packet accordingly. Then, it waits for ACK packet from the nano-controller. If the nano-node receives the corresponding ACK packet, this transmission ends. If nano-node does not receive the ACK for a timeout or receives the Negative ACKnowledgement (NACK) from the nano-controller, it will retransmit the data packet in the next time frame. The timeout equals to $2B_{ACK}T_{s}$.
- In particular, when a nano-node does not have data to be sent to the nano-controller in its SAS, it can send a Clear To Receive (CTR) packet to the nano-controller. After receiving the CTR, the nano-controller can send data to the nano-node for specific function updating, time synchronization, etc.

### 4.3. Transmission Scheme in Distributed Nano-Networks

In distributed nano-networks, the communications among nano-nodes are self-organized. Due to the differences between centralized and distributed nano-networks, the transmission schemes of nano-nodes in those two nano-networks have several differences. The detailed transmission operations of nano-nodes in distributed nano-networks are as follows:

- In the initial stage, the operations of nano-nodes in distributed nano-networks are identical with centralized nano-networks, i.e., allocating the first time slots as RBSs, one time slot as SAS chosen by their IDs, and the other time slots as SSs.
- In RBSs, on the one hand, nano-nodes can run time synchronization methods or receive time packets from special nano-controllers to synchronize. On the other hand, nano-nodes can do neighbor discovering process by broadcasting small packets with their own IDs; any nano-node who receives the packets will record the corresponding nano-nodes as its neighbors. However, nano-nodes do not need to execute the above procedures in every time frame.
- In SASs, nano-nodes start to listen to the channel and receive data from other nano-nodes. If the data packet is received correctly, the nano-node will reply an ACK packet. If not, it will reply a NACK packet. The nano-nodes only receive packets with sufficient energy, i.e., the energy of nano-nodes exceeds the energy of receiving one data packet and transmitting one ACK/NACK packet. Otherwise, they will wait for the next time frame and harvest energy from the environment.
- When a nano-node needs to send or forward a packet to a specific nano-node, it firstly calculates the SAS of the receiving nano-node according to Equation (1), and then it turns the corresponding SS into transmitting slot to send the packet. If the transmitting nano-node receives ACK from the receiving nano-node, the transmission ends. If it does not receive the ACK for a timeout or receive NACK from the receiver, the data packet will be retransmitted in the next time frame. As in centralized nano-networks, The nano-nodes only begin the transmission with sufficient energy.
- As in centralized nano-network, nano-nodes in distributed nano-networks also can harvest energy and collect data from the environment in SSs.

However, it could be difficult to design a routing protocol over SSA-MAC because nano-nodes must wait for the opportunity slot of a neighbor to retransmit its packet. Furthermore, a tradeoff between energy consumption and successful communication must be taken into consideration.

However, the opportunistic routing, and routing algorithms in Delay-Tolerant networking (DTN) could help the design of routing protocols based on SSA-MAC. Although they cannot be used in nano-networks directly due to the resources constraints of nano-nodes and peculiarities of THz band, these routing algorithms can provide directions for the protocols design. We are now working on this.

In traditional wireless sensor networks (WSNs), working–sleeping scheduling schemes are used to prolong the lifetime of sensors, but also cause opportunistic node connections due to the intermittent communication mode, which is quite similar to SSA-MAC. In WSNs, opportunistic routing technologies are proposed to address the intermittent communication issues of sensors, and the exponential growth of the number of wireless connected devices by providing multi routing paths and to improve the reliability of networks [41,42]. The authors of [43] proposed an opportunistic routing algorithm that uses predictive retransmissions to maximize the probability of success for each transmission, which could be used for the design of routing protocols based on SSA-MAC.

Moreover, nano-networks are not expected to achieve high transmission bit-rates [19]. Hence, some routing protocols in DTN could help to address delay and lack of continuous network connectivity [44]. Unlike the traditional networks, such as TCP-based Internet, DTN are often subject to high latency and intermittent connectivity. The situation is quite similar to in SSA-MAC: the nano-nodes have to harvest energy and wait for their own slots, which could also cause latency and intermittent connectivity. For example, a double Q-learning routing algorithm is proposed for DTN to improve the connective efficiency, while balancing the routing performance and cost [45]. In [46], reliable energy-aware routing protocol for DTN is proposed to address the energy depletion of nodes.

### 4.4. Packet Format

**Figure 6:**
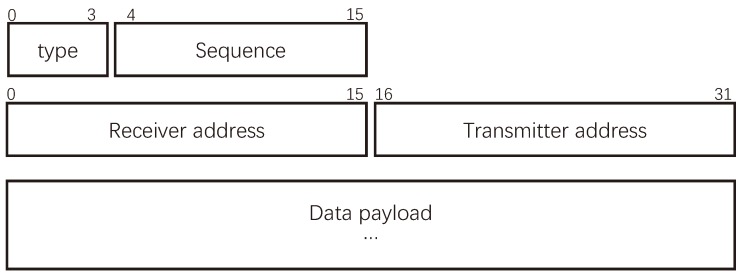
The packet format.

Due to the peculiarities of SSA-MAC, the format of the packets should be redesigned. In the SSA-MAC, the data packet can be classified into broadcast data packet and unicast data packet. The broadcast data packet is sent by the nano-controller and is used for information and function updating, and time synchronization, etc. The unicast data packet is used for delivering collected data information. The packet format of data packet is present in Figure 6, where type refers to the type of data packet, “0001” represents the broadcast data packet and “0010” represents the unicast data packet. The Sequence is the sequence number of packets and occupies 12 bits. The address of receiver and transmitter occupy 16 bits, respectively.

Moreover, there are three control packets in SSA-MAC, namely ACK, NACK and CTR. When a nano-node or nano-controller receives a data packet correctly, it will reply an ACK packet. Otherwise, it will reply a NACK packet. In centralized nano-networks, if nano-nodes do not have data to be sent in their own SASs, they will send CTR packets to the nano-controller. The nano-controller can send updating packets to the corresponding nano-nodes after receiving the CTR packets. The formats of control packets are identical with those of data packets, but have no data payload. The type indicates the types of control packets, in the SSA-MAC, “0100” refers to the ACK packet, “1000” refers to the NACK packet and “1001” refers to the CTR packet.

## 5. Performance Analysis

In this section, the performance of SSA-MAC protocol is analyzed, including the energy consumption, packet delay and throughput.

### 5.1. Energy Consumption

Due to the size limitation of nano-node, its energy storage is expected to be very small. Hence, the energy efficiency of MAC protocol is critical in nano-networks. At present, The energy consumption of nano-processors is still unknown. Therefore, we concentrate on the energy consumption that would be spent in the communication part.

#### 5.1.1. Energy Consumption of Transmitting Packets

The transmitting packet operations in centralized and distributed nano-networks are identical, i.e., the nano-node sends the packet directly in the corresponding time slot and waits for the ACK packet. The transmission procedure could fail when: (i) the packet is not received correctly by the receiver due to collisions or other errors; or (ii) the ACK packet is not received by the transmitter correctly due to collisions or other errors. The probability of the above two cases are as follows:

(8) $$p_{1}=1-p_{Data}+p_{Data}p_{col},$$

(9) $$p_{2}=p_{Data}\left(1-p_{col}\right)\left(1-p_{ACK}\right),$$

where $p_{Data}$ and $p_{ACK}$ are the probabilities of no symbol error occurs during data and ACK packets transmissions, respectively. $p_{col}$ is the probability of collision, which relates to the number of nano-nodes in one time slot. We consider the collisions follow the exponential distribution. Then, $p_{col}$ can be obtained by

(10) $$p_{col}=1-\exp\left(-\lambda_{t}\frac{N_{node}}{k}\frac{T_{i}}{T_{s}}\right),$$

where exp refers to the exponential function, $\lambda_{t}$ is the sending packet intensity of nano-nodes, and $T_{i}$ is the signal integration time, which is ten times of pulse duration $T_{p}$ [19]. Moreover, the probability of transmitting the data packet successfully can be expressed as follows:

(11) $$p_{3}=p_{Data}p_{ACK}\left(1-p_{col}\right).$$

On the other hand, the energy consumption of the above three cases can be obtained by

(12) $$E_{1}=E_{Data}^{T},$$

(13) $$E_{2}=E_{Data}^{T}+E_{ACK}^{R},$$

(14) $$E_{3}=E_{Data}^{T}+E_{ACK}^{R},$$

where $E_{1}$ is the consumed energy when the data packet is not sent correctly and the ACK packet is not received, $E_{2}$ is the consumed energy when the ACK packet is received incorrectly or the NACK is received by the transmitter, and $E_{3}$ is the consumed energy when the packet is transmitted successfully.

Above all, the energy consumption of transmitting a packet successfully can be obtained as follows [19]:

(15) $$E_{Trans}=\frac{1}{p_{3}}\left(p_{1}E_{1}+p_{2}E_{2}+p_{3}E_{3}\right)$$

Substituting Equations (8)–(14) into Equation (15), the energy consumption of one packet can be rewritten as follows:

(16) $$\begin{array}{cc} E_{Trans}= & \frac{1}{p_{Data}p_{ACK}\left(\exp\left(-\lambda_{t}\frac{N_{node}}{k}\frac{T_{i}}{T_{s}}\right)\right)}\left(\left(1-p_{Data}+p_{Data}\left(1-\exp\left(-\lambda_{t}\frac{N_{node}}{k}\frac{T_{i}}{T_{s}}\right)\right)\right)E_{Data}^{T}+\right.\\ & p_{Data}\exp\left(-\lambda_{t}\frac{N_{node}}{k}\frac{T_{i}}{T_{s}}\right)\left(1-p_{ACK}\right)\left(E_{Data}^{T}+E_{ACK}^{R}\right)+\\ & \left.p_{Data}p_{ACK}\exp\left(-\lambda_{t}\frac{N_{node}}{k}\frac{T_{i}}{T_{s}}\right)\left(E_{Data}^{T}+E_{ACK}^{R}\right)\right). \end{array}$$

#### 5.1.2. Energy Consumption of Receiving Packets

In both centralized and distributed nano-networks, nano-nodes can receive the broadcast packet from nano-controllers to update the information; the energy consumption of this operation is denoted as $E_{B}$.

In centralized nano-networks, nano-nodes can send CTRs to the nano-controller when there is no data to be sent to the nano-controller in their SASs. Then, the nano-controller can send updating packets to the nano-nodes. The operations are quite similar to the transmitting packet process, as well as the calculations of energy consumption. Hence, we do not repeat the calculations here. Similarly, the energy consumption of receiving update packet from the nano-controller in nano-nodes’ SASs can be expressed as

(17) $$\begin{array}{cc} E_{Rec}^{U}= & \frac{1}{p_{CTR}p_{Data}\left(\exp\left(-\lambda_{t}\frac{N_{node}}{k}\frac{T_{i}}{T_{s}}\right)\right)}\left(\left(1-p_{CTR}+p_{CTR}\left(1-\exp\left(-\lambda_{t}\frac{N_{node}}{k}\frac{T_{i}}{T_{s}}\right)\right)\right)E_{CTR}^{T}+\right.\\ & p_{CTR}\exp\left(-\lambda_{t}\frac{N_{node}}{k}\frac{T_{i}}{T_{s}}\right)\left(1-p_{Data}\right)\left(E_{CTR}^{T}+E_{Data}^{U}\right)+\\ & \left.p_{CTR}p_{Data}\exp\left(-\lambda_{t}\frac{N_{node}}{k}\frac{T_{i}}{T_{s}}\right)\left(E_{CTR}^{T}+E_{Data}^{U}\right)\right), \end{array}$$

where $p_{CTR}$ is the probability that no symbol error occurs during the CTR packet transmission, $E_{CTR}^{T}$ refers to the energy consumption of transmitting a CTR packet, and $E_{Data}^{U}$ refers to the energy consumption of receiving an updating data packet.

However, in distributed nano-networks, nano-nodes also receive packets from surrounding nano-nodes. Similar to the energy consumption of transmitting a packet, the consumed energy in distributed nano-networks for receiving a packet can be obtained by

(18) $$E_{Rec}^{Dis}=\frac{1}{p_{3}}\left(p_{1}E_{Data}^{R}+p_{2}\left(E_{Data}^{R}+E_{ACK}^{T}\right)+p_{3}\left(E_{Data}^{R}+E_{ACK}^{T}\right)\right),$$

where $E_{ACK}^{T}$ is the consumed energy of sending an ACK packet and $E_{Data}^{R}$ is the consumed energy of receiving a data packet from the other nano-nodes. Combining with Equations (8)–(11), the consumed energy of receiving a packet in distributed nano-networks $E_{Rec}^{Dis}$ can be rewritten as follows:

$$\begin{array}{c} E_{Rec}^{Dis}=\frac{1}{p_{Data}p_{ACK}\left(\exp\left(-\lambda_{t}\frac{N_{node}}{k}\frac{T_{i}}{T_{s}}\right)\right)}\left(\left(1-p_{Data}+p_{Data}\left(1-\exp\left(-\lambda_{t}\frac{N_{node}}{k}\frac{T_{i}}{T_{s}}\right)\right)\right)E_{Data}^{R}+\right. \end{array}$$

(19) $$\begin{array}{c} p_{Data}\exp\left(-\lambda_{t}\frac{N_{node}}{k}\frac{T_{i}}{T_{s}}\right)\left(1-p_{ACK}\right)\left(E_{Data}^{R}+E_{ACK}^{T}\right)+\\ \left.p_{Data}p_{ACK}\exp\left(-\lambda_{t}\frac{N_{node}}{k}\frac{T_{i}}{T_{s}}\right)\left(E_{Data}^{R}+E_{ACK}^{T}\right)\right). \end{array}$$

### 5.2. Packet Delay

In the proposed SSA-MAC protocol, if the nan-node receives a NACK or does not receive the ACK packet from the nano-controller or receiving nano-node, it will retransmit the packet in the next time frame. Hence, for both kinds of nano-networks, the packet transmission delay without energy restriction can be expressed as

(20) $$D_{Trans}=\frac{T_{frame}}{p_{3}}\left(p_{1}+p_{2}+p_{3}\right).$$

Substituting Equations (8)–(11) into Equation (20), the packet transmission delay without energy restriction can be rewritten as

(21) $$\begin{array}{cc} D_{Trans}= & \frac{T_{frame}}{p_{Data}p_{ACK}\left(\exp\left(-\lambda_{t}\frac{N_{node}}{k}\frac{T_{i}}{T_{s}}\right)\right)}\left(\left(1-p_{Data}+p_{Data}\left(1-\exp\left(-\lambda_{t}\frac{N_{node}}{k}\frac{T_{i}}{T_{s}}\right)\right)\right)+\right.\\ & \left.p_{Data}\exp\left(-\lambda_{t}\frac{N_{node}}{k}\frac{T_{i}}{T_{s}}\right)\left(1-p_{ACK}\right)+p_{Data}p_{ACK}\exp\left(-\lambda_{t}\frac{N_{node}}{k}\frac{T_{i}}{T_{s}}\right)\right). \end{array}$$

However, nano-nodes are also limited by energy; they can only transmit or receive packets with sufficient energy. Hence, there exists energy harvesting delay. For centralized nano-networks, the energy harvesting delay for a packet can be expressed as follows:

(22) $$D_{harv}^{Cen}=\frac{E_{Trans}+\alpha_{1}\left(E_{B}+E_{Rec}^{U}\right)}{S_{harv}},$$

where $\alpha_{1}$ is the frequency of receiving updating packets from nano-controller in centralized nano-networks, since updating operations are not necessary in every time frame. For distributed nano-networks, the energy harvesting delay for a packet can be obtained by

(23) $$D_{harv}^{Dis}=\frac{\beta E_{Trans}+E_{Rec}^{Dis}+\alpha_{2}E_{B}}{S_{harv}},$$

where β is the average number of transmitting packets in one time frame of nano-node. $\alpha_{2}$ is the frequency of receiving updating packets from other nano-nodes in distributed nano-networks. Moreover, since the propagation time is too small compared to the transmission delay and energy harvesting delay, it is not considered in the model.

### 5.3. Throughput

The throughput of nano-node depends on several parameters, including energy harvesting speed, energy consumption speed and packet transmission delay. When energy harvesting speed is larger than energy consumption speed, the throughput is mainly decided by packet transmission delay. Otherwise, the throughput is mainly decided by energy harvesting speed. Hence, the achievable throughput of a nano-node can be obtained by

(24) $$T_{put}=\min\left(\frac{B_{Data}}{D_{harv}},\frac{B_{Data}}{D_{trans}}\right)$$

where $D_{harv}$ is the energy harvesting delay, in centralized nano-networks, $D_{harv}=D_{harv}^{Cen}$, in distributed nano-networks, $D_{harv}=D_{harv}^{Dis}$.

## 6. Performance Evaluation

To investigate the performance of SSA-MAC with different parameters, extensive simulations were conducted in this section based on the performance analysis in Section 4. Moreover, we compared the SSA-MAC with PHLAME, RIH-MAC and EEWNSN MAC protocols. In PHLAME, nano-nodes need to send handshake packets to the receivers to check the nano-node availability and exchange symbol rates before transmitting data packets. In RIH-MAC, the receiving nano-nodes keep sending RTR packets to surrounding nano-nodes periodically. When the surrounding nano-nodes receive the RTR packet and have enough energy, they will compete to send data packets to the corresponding receiving nano-node. In EEWNSN, nano-nodes need to communicate with the nano-controller to allocate transmitting time slots. Therefore, it cannot be used in distributed nano-networks. The transmission schemes in centralized and distributed nano-networks are different, thus the performances of these two nano-network structures were investigated separately.

### 6.1. Performance Metrics and Parameters Setting

To unify the evaluation criteria of the performance evaluations, we considered two performance metrics: (i) energy consumption per bit, which can reflect the energy efficiency of protocols and bigger energy consumption per bit indicates lower energy efficiency; and (ii) nano-node achievable throughput. Since the throughput of nano-node depends on the packet transmission delay and energy harvesting delay according to Equation (24), it also can be used to evaluate the delay of the whole nano-network. Bigger nano-node achievable throughput indicates lower packet delay.

The values of parameters used in the simulations are listed in Table 1. The values of TS-OOK (including pulse duration, symbol duration, pulse energy, pulse integration time and packet error rate) were referred to [19,32]. The values of network structure (including nano-nodes density, transmission range, packet size and updating coefficient) were referred to [20,39]. The node-density was set from 0.1 to 2.5 nano-node/${\mathrm{mm}}^{2}$, thus the number of nano-nodes being evaluated ranged from 31 to 785. A nano-piezoelectric system based on zinc-oxide was implemented as the energy harvesting system [12,25,47]. The performance of different MAC protocols was investigated with different nano-node densities and energy harvesting speeds.

### 6.2. Performance Comparison of Different MAC Protocols

We compared the proposed SSA-MAC protocol with PHLAME, RIH-MAC and EEWNSN MAC protocols in terms of energy consumption per bit, and achievable throughput in centralized nano-networks.

#### 6.2.1. Performance in Centralized Nano-Networks

**Figure 7:**
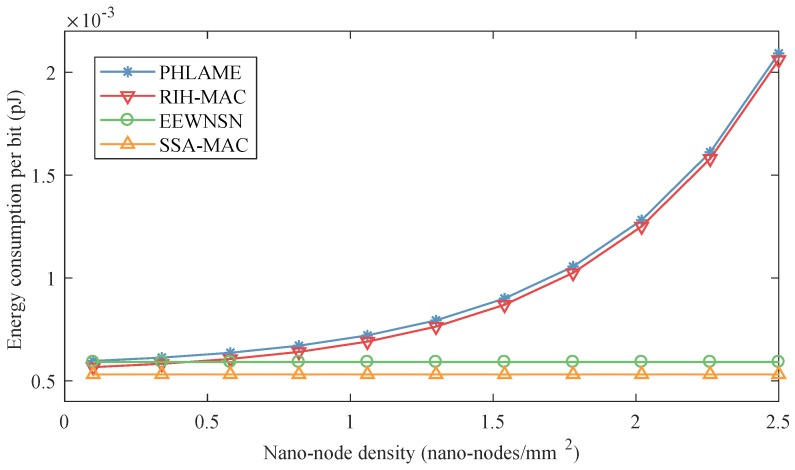
Energy consumption per bit of different MAC protocols as a function of nano-node density in centralized nano-networks.

Figure 7 shows the energy consumption per bit of different MAC protocols with different nano-node densities in centralized nano-networks, in these simulations, energy harvesting speed was set to 3 pJ/s. It can be concluded from Figure 7 that:

(i) With the increase of nano-node density, the energy consumption per bit of PHLAME and RIH-MAC increases, and their growth rates become bigger and bigger, e.g., from 1 nano-node/${\mathrm{mm}}^{2}$ to 1.5 nano-nodes/${\mathrm{mm}}^{2}$, the energy consumption per bit of PHLAME increases $0.2\times 10^{-3}$ pJ. However, it increases $0.8\times 10^{-3}$ pJ when the nano-node density increases from 2 nano-nodes/${\mathrm{mm}}^{2}$ to 2.5 nano-nodes/${\mathrm{mm}}^{2}$. The reason is that the quantity of nano-nodes increases with the nano-node density, which results in the increase of collision probability during transmissions according to Equation (10). Nano-nodes have to retransmit the data packet when collisions occur, which increases the energy consumption for one packet, especially when nano-node density is large.

(ii) However, the energy consumption per bit of EEWNSN and SSA-MAC does not change much with the increase of nano-node density because, in these two MAC protocols, nano-nodes can transmit the data packet during their own transmitting time slots, and, hence, collisions seldom happen.

(iii) When nano-node density is small (less than 0.5 nano-nodes ${\mathrm{mm}}^{2}$), the order of energy consumption per bit of the four MAC protocols is PHLAME > EEWNSN > RIH-MAC > SSA-MAC. The reason PHLAME has the highest energy consumption per bit is that the nano-nodes will check the availability and exchange symbol rates with the receiving nano-nodes by handshake process before transmitting the packets, which needs extra energy and could introduce more collisions. The reasons the energy consumption per bit of EEWNSN is larger than RIH-MAC are that, on the one hand, the nano-nodes need to communicate with the nano-controllers to allocate the transmitting time slot in EEWNSN, which needs extra energy, and, on the other hand, when the nano-node density is small, the collisions among nano-nodes are not strong in RIH-MAC. However, in SSA-MAC, nano-nodes do not need handshake with other nano-nodes or communicate with the nano-controller to allocate time slot. In addition, nano-nodes can transmit data packets during their own time slots. Therefore, it has the lowest energy consumption per bit.

(iv) When the nano-node density is big (more than 0.5 nano-nodes ${\mathrm{mm}}^{2}$), the order of energy consumption per bit of the four MAC protocols is PHLAME > RIH-MAC > EEWNSN > SSA-MAC. The main reason that the performance of EEWNSN becomes better than RIH-MAC is that, with the increase of nano-nodes, the collision probabilities of PHLAME and RIH-MAC increase drastically, which results in more packet retransmissions.

**Figure 8:**
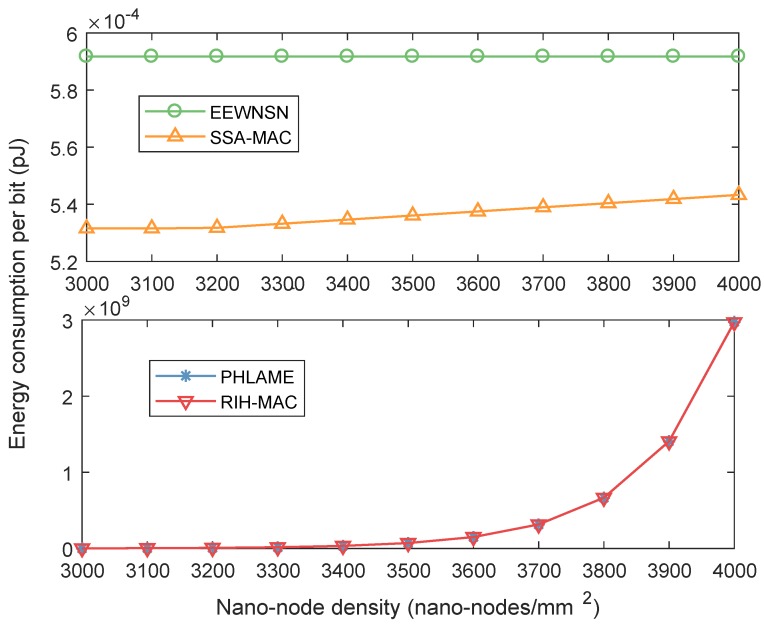
Energy consumption per bit of different MAC protocols with much higher nano-node density in centralized nano-networks.

In fact, according to Equations (3), (6) and (7), a time frame can be divided into tens of thousands of time slots. Hence, nano-nodes in SSA-MAC can still maintain a good performance when nano-node density is very large. The performance of different protocols with much higher nano-node density is investigated in Figure 8, where the nano-node density is set from 3000 to 4000 nano-nodes/${\mathrm{mm}}^{2}$, i.e., the number of nano-nodes is up to one million. It can be observed that the energy consumption per bit of PHLAME and RIH-MAC is much higher than EEWNSN and SSA-MAC. Comparing Figure 7 and Figure 8, the EEWNSN and SSA-MAC can still maintain good performance when nano-node density is very large, since nano-nodes in these two protocols can transmit in their own time slots. The proposed SSA-MAC performs better than EEWNSN, since it does not need to communicate with the nano-controller to allocate time slots. However, when nano-node density exceeds 3200 nano-nodes/${\mathrm{mm}}^{2}$, the energy consumption per bit of SSA-MAC increase with the node density. Since, in this simulation, a time frame can only be divided into one million time slots according to Equations (3), (6) and (7), some nano-nodes have to transmit in the same slot, which results in collisions. Nevertheless, we can enlarge the length of time frame to eliminate the collisions.

Moreover, considering the nano-node density adopted by Jornet et al. [19] (PHLAME), Mohrehkesh and Weigle [20] (RIH-MAC) and Rikhtegar et al. [32] (EEWNSN), as well as for the convenience of performance comparisons for different MAC protocols, the nano-node density in the next simulations was still set from 0.1 to 2.5 nano-nodes/${\mathrm{mm}}^{2}$.

**Figure 9:**
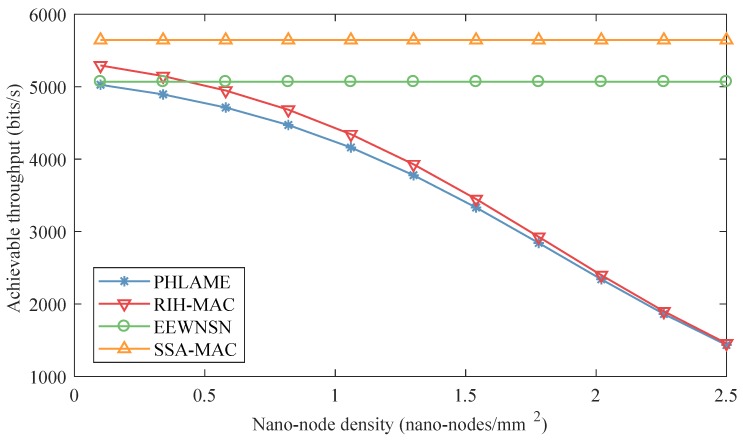
Achievable throughput of different MAC protocols as a function of nano-node density in centralized nano-networks.

The achievable throughput of different MAC protocols as a function of nano-node density in centralized nano-networks is presented in Figure 9. In these simulations, energy harvesting speed was set to 3 pJ/s. The achievable throughputs of PHLAME and RIH-MAC decrease drastically with nano-node density, e.g., the achievable throughput when nano-node density is 2.5 nano-nodes/${\mathrm{mm}}^{2}$ is only 30% when density is 0.5 nano-nodes/${\mathrm{mm}}^{2}$. The reason is that the collision probabilities of the two protocols increase with nano-node density according to Equation (10). However, the achievable throughputs of EEWNSN and SSA-MAC do not change much when the nano-node density increases. In EEWNSN, the nano-nodes can communicate with the nano-controller to allocate their own transmitting time slot. In SSA-MAC, a time frame will be divided into several time slots by the number of nano-nodes and peculiarities of TS-OOK, where nano-nodes can transmit/receive in their own time slot allocated by their ID. Therefore, collisions in these two protocols seldom happen according to Equation (10), i.e., nano-nodes do not need to cost extra resources to retransmit the packet, and can maintain a good performance. The achievable throughput of SSA-MAC is 10% higher than EEWNSN, because the nano-nodes in the SSA-MAC do not need to communicate with the nano-controller to allocate the transmitting time slot. Comparing Figure 7 and Figure 9, it can be observed that the achievable throughput of nano-node is negatively correlated with energy consumption per bit. That is, the achievable throughput of nano-node is mainly decided by the energy harvesting speed. Therefore, to design more advanced energy harvesting systems and more energy-efficient communication protocols are the keys to improve the performance of nano-networks.

**Figure 10:**
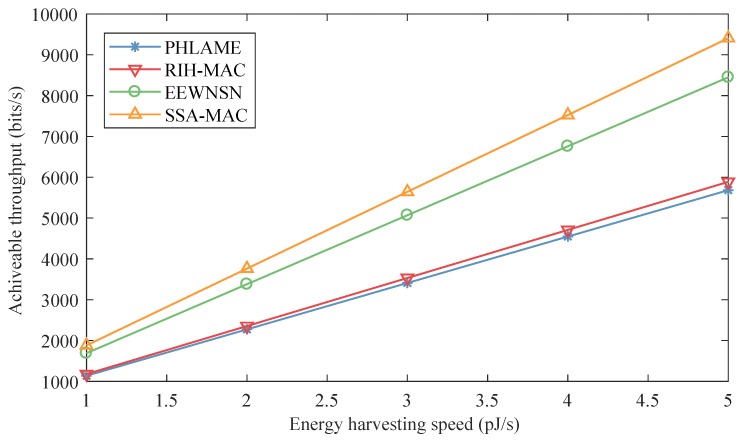
Achievable throughput of different MAC protocols as a function of energy harvesting speed in centralized nano-networks.

Moreover, the achievable throughput was investigated with different energy harvesting speeds, and the results are shown in Figure 10. In these simulations, the nano-node density was set to 1.5 nano-nodes/${\mathrm{mm}}^{2}$. From the above two groups of simulations, we can conclude that the throughput of nano-node is mainly affected by the energy harvesting speed. It also can be verified in Figure 10 that the achievable throughputs of all MAC protocols increase with the energy harvesting speed. The proposed SSA-MAC has the highest throughput. Furthermore, with the increase of energy harvesting speed, the throughput gaps between SSA-MAC and other MAC protocols also increase, e.g., when the energy harvesting speed is 3 pJ/s, the achievable throughput of SSA-MAC is almost 600 bits/s, 2400 bits/s and 2500 bits/s higher than EEWNSN, RIH-MAC and PHLAME protocols, respectively. However, when the energy harvesting speed is 5 pJ/s, the achievable throughput of SSA-MAC is almost 1000 bits/s, 3450 bits/s and 3600 bits/s higher than EEWNSN, RIH-MAC and PHLAME protocols, respectively. This also indicates that the proposed SSA-MAC has better energy efficiency than other MAC protocols.

#### 6.2.2. Performance in Distributed Nano-Networks

In distributed nano-networks, the communications among nano-nodes are self-organized. However, in the EEWNSN MAC protocols, nano-nodes need to communicate with the nano-controllers to allocate the transmitting time slot; it cannot be applied in distributed nano-networks. Therefore, SSA-MAC was compared with PHLAME and RIH-MAC protocols in terms of energy consumption per bit and achievable throughput. Additionally, different from the centralized nano-networks, nano-nodes need to consume energy to receive from surrounding nano-node in distributed nano-networks. We considered the receiving energy as one-tenth the transmitting energy [39].

**Figure 11:**
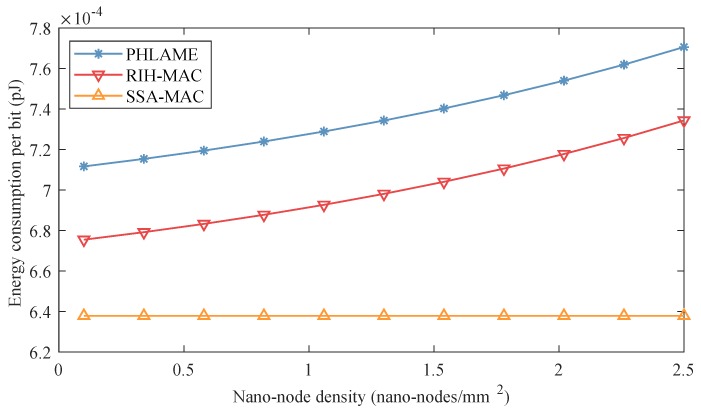
Energy consumption per bit of different MAC protocols as a function of nano-node density in distributed nano-networks.

In Figure 11, the energy consumption per bit of different MAC protocols is presented as a function of nano-node density. In these simulations, energy harvesting speed was set to 3 pJ/s. We can get similar conclusions as in centralized nano-networks that: (i) With the increase of nano-node density, the energy consumption per bit of PHLAME and RIH-MAC protocols also increases. The reason is that the increase of nano-node quantity enlarges the collision probability, which would increase the packet retransmission. (ii) In SSA-MAC, nano-nodes can transmit packets in their own SASs, and, thus, it can still maintain a good performance when nano-node density increases. (iii) The energy consumption per bit in distributed nano-networks is higher than it is in centralized nano-networks when nano-node density is small. For example, when nano-node density is 0.5 nano-node/${\mathrm{mm}}^{2}$, the energy consumption per bit in distributed nano-networks is near 10% higher than it in centralized nano-networks because, in distributed nano-network, nano-nodes also need to consume energy to receive data packets from surrounding nano-nodes, but in centralized nano-networks, all the nano-nodes send data packets to the nano-controller. (iv) However, for PHLAME and RIH-MAC protocols, when nano-node density is large, the energy consumption per bit in distributed nano-networks becomes smaller than it in centralized nano-networks, e.g., when nano-node density is 2.5 nano-nodes/${\mathrm{mm}}^{2}$, the energy consumption per bit in distributed nano-networks is 60% less than it is in centralized nano-networks. Since the transmission range of nano-controller is larger than nano-node, there will be more nano-nodes in centralized nano-networks when nano-node density increases, which makes it easier to cause collisions, resulting in more retransmissions.

**Figure 12:**
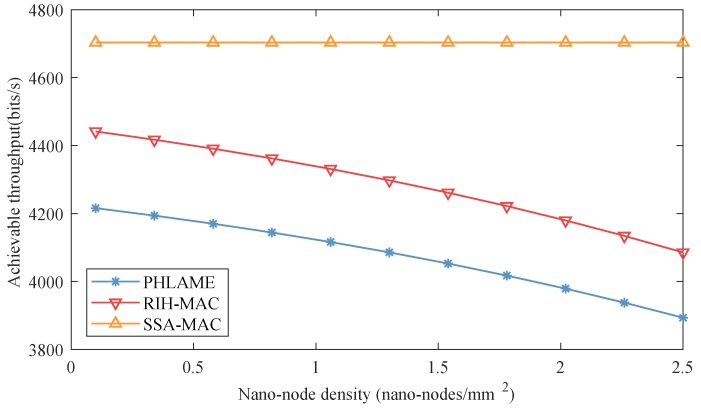
Achievable throughput of different MAC protocols as a function of nano-node density in distributed nano-networks.

The achievable throughputs of different MAC protocols are presented in Figure 12 as a function of nano-node density. In these simulations, energy harvesting speed was set to 3 pJ/s. Combining with Figure 11, it can be observed that the energy consumption per bit and the achievable throughput change in the opposite direction with nano-node density. This indicates that the achievable throughput of nano-node is mainly affected by energy harvesting speed. For the SSA-MAC protocol, the achievable throughput in distributed nano-networks is smaller than it is in centralized nano-networks, since nano-nodes also need to consume energy when receiving data packets from surrounding nano-nodes. However, for PHLAME and RIH-MAC protocols, when the nano-node density is large, the achievable throughput of distributed nano-networks is larger than it is in centralized nan-networks. Since the transmission range of nano-controller is bigger than nano-node, there will be more nano-nodes causing collisions in centralized nano-networks. Moreover, with different nano-node densities, the achievable throughput of SSA-MAC protocol is always higher than PHLAME and RIH-MAC protocols.

**Figure 13:**
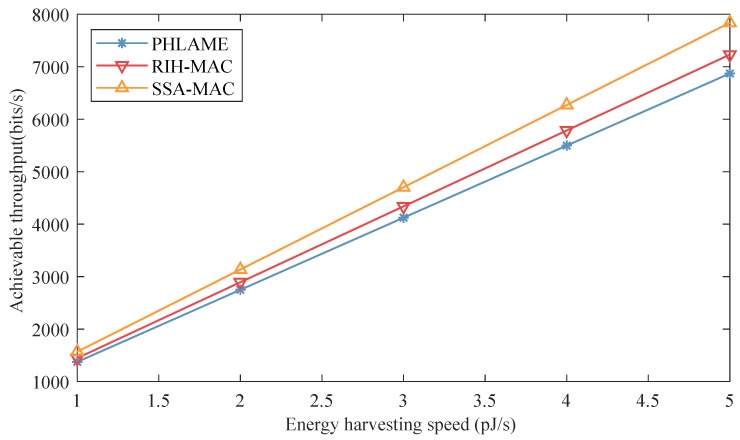
Achievable throughput of different MAC protocols as a function of energy harvesting speed in distributed nano-networks.

The achievable throughputs of different MAC protocols were also investigated with different energy harvesting speeds in distributed nano-networks. The results are shown in Figure 13. The nano-node density used in these simulations was set to 1.5 nano-nodes/${\mathrm{mm}}^{2}$. As mentioned above, the achievable throughput of nano-node is mainly decided by the energy harvesting speed. It also can be verified in Figure 13, where the achievable throughput increases with the energy harvesting speed. Moreover, it also can be observed that SSA-MAC performs better than the other two protocols, since it does not need a handshake process or send RTR packet before transmitting packets.

## 7. Conclusions

In this paper, a slot self-allocation based MAC protocol is proposed for energy harvesting nano-networks. In the protocol, a time frame is divided into several time slots, and nano-nodes can allocate a self-allocated slot by their own ID. Two transmission schemes are designed for different nano-network structures. In centralized nano-networks, nano-nodes can only send packets to the nano-controller in their own self-allocated slot. In distributed nano-networks, nano-nodes only can receive packets from the surrounding nano-nodes in their own self-allocated slot. This protocol can effectively avoid data retransmissions caused by collisions and can improve the energy efficiency of nano-networks.

Simulations were conducted to compare the proposed SSA-MAC protocol with PHLAME, RIH-MAC and EEWNSN MAC protocols in terms of energy consumption per bit and achievable throughput of nano-node with different nano-node densities and energy harvesting speeds. Comprehensively, our proposed SSA-MAC protocol has the best performance.

## Figures and Tables

**Table 1:** Simulation parameters.

Parameter	Values
Nano-node density	[0.1–2.5] nodes/${\mathrm{mm}}^{2}$
Pulse duration $T_{p}$	100 fs
Symbol duration $T_{s}$	10 ps
Pulse energy	1000 aJ
Transmission range of nano-node	5 mm
Transmission range of nano-controller	10 mm
Radius of the network $T_{p}$	10 mm
Data packet size $B_{Data}$	100 bytes
Control packet size	6 bytes
Energy harvesting speed $E_{harv}$	[1–5] pJ/s
Time frame $T_{frame}$	$\left(E_{Data}^{T}+E_{ACK}^{R}\right)/S_{harv}$
Ratio of symbol “1” in one packet $\eta_{Data}$, $\eta_{ACK}$	0.5
Pulse integration time $T_{i}$	1000 fs
Packet error rate	$10^{-3}$
Updating coefficient $\alpha_{1}$, $\alpha_{2}$	$10^{-2}$
Transmitting time β	1
